# Bombesin antagonists inhibit proangiogenic factors in human experimental breast cancers

**DOI:** 10.1038/sj.bjc.6601404

**Published:** 2004-01-06

**Authors:** A M Bajo, A V Schally, K Groot, K Szepeshazi

**Affiliations:** 1 Endocrine Polypeptide and Cancer Institute, Veterans Affairs Medical Center, New Orleans, LA 70112-1262, USA; 2 Department of Medicine, Section of Experimental Medicine, Tulane University School of Medicine, New Orleans, LA 70112-2699, USA

**Keywords:** cancer therapy, breast cancer, angiogenesis factors

## Abstract

The overexpression of angiogenic factors such as vascular endothelial growth factor (VEGF), fibroblast growth factor (FGF) and insulin-like growth factors (IGFs) plays a role in the migration and proliferation of endothelial cells in many cancers. Consequently, we investigated the effects of bombesin/gastrin-releasing peptide (GRP) antagonists on the expression of these angiogenic factors, the activities of matrix metalloproteinases (MMPs)-2 and -9, as well as the vascular density in MDA-MB-435 human oestrogen-independent breast cancers. Nude mice bearing orthotopic xenografts of MDA-MB-435 breast cancers were treated with bombesin/GRP antagonists for 6 weeks. Daily administration of 20 *μ*g of RC-3095 or 10 *μ*g of RC-3940-II significantly decreased the weight of MDA-MB-435 cancers by 44 and 53%, respectively. The inhibition of tumour growth was associated with a substantial reduction in the expression of mRNA and protein levels of basic fibroblast growth factor (bFGF), IGF-II and VEGF-A in the tumours. Both bombesin/GRP antagonists significantly decreased the vessel density of the tumours by about 37%, as shown by immunohistochemical detection of vessels on tumour slides. Gelatinolytic activities, detected by zymography, revealed a 33–46% reduction in MMP-9 activity after the treatment with either antagonist. *In vitro* studies revealed that MDA-MB-435 cells secrete bFGF, IGF-II and VEGF-A, and the secretion of these factors is inhibited by RC-3095 and RC-3940-II. This study demonstrates the antiangiogenic effect of bombesin/GRP antagonists RC-3095 and RC-3940-II, and underscores their possible therapeutic application for treatment of breast cancers.

Breast cancer is the second most frequent cause of cancer-related deaths among women in the Western world (Jemal *et al*, 2002). The use of surgical resection combined with chemotherapy and/or radiotherapy and early diagnostic techniques has led to a substantial reduction in deaths from breast cancer over the last decade. However, many patients still develop recurrent or metastatic breast cancer. Therefore, new therapeutic strategies are needed, especially for advanced oestrogen-independent tumours (Schally and Comaru-Schally, 2003).

The insulin-like growth factors (IGFs) and their binding proteins are known to be involved in the growth and maintenance of the neoplastic breast tissue. The IGFs primarily exert their biologic effects through binding to the IGF-I receptor (IGF1R), which initiates an intracellular signalling cascade, including the Ras/Raf/MAP kinase and PI 3 kinase pathways, to stimulate mitogenesis and differentiation. Since IGF-1 receptor requires an activation by the ligand, in contrast to other transmembrane growth factor receptors, the regulation of the ligand would be a relevant treatment in breast cancer (reviewed in Gross and Yee, 2003).

Neovascularisation, the process by which new blood vessels are formed from pre-existing host vasculature, is an essential requirement for tumour growth and metastasis (Folkman, 1990). This complex process involves proteolysis of basement membrane, endothelial cell migration, proliferation and matrix remodelling. Several growth factors such as vascular endothelial cell growth factor (VEGF), basic fibroblast growth factor (bFGF) and IGF-I and -II contribute either alone or in combination to the secretion of metalloproteinases (MMPs), which results in the degradation of basement membrane in microvessel walls, and the formation of new blood vessels (Kandel *et al*, 1991; Grant *et al*, 1993; Neufeld *et al*, 1999; Lee *et al*, 2000; Yan *et al*, 2000). The disruption of the vascular basement membrane may facilitate extravasation of endothelial cells, leading to the formation of neovascular sprouts, as well as intravasation of tumour cells into the vessels. The overexpression of the members of VEGF, FGF and IGF families, as well as their receptors, has been reported in a significant percentage of breast tumours (Peyrat *et al*, 1992; Brown *et al*, 1995; Yee, 1998). Moreover, the overexpression of human epidermal growth factor receptor-2 (ErbB-2/HER-2), which is associated with poor survival in several human malignancies including breast cancers, is closely correlated with increased angiogenesis and expression of VEGF (reviewed in Kumar and Yarmand-Bagheri, 2001). In addition, the ability to degrade the extracellular matrix is an important determinant of the invasive phenotype. One group of proteolytic enzymes associated with tumour invasion is the MMP family (Nagase and Woessner, 1999). Most of them are secreted as pro-enzymes, which are extracellularly processed to generate their active forms. Gelatinases A (MMP-2) and B (MMP-9) can both degrade type IV collagen of basement membranes, the first barrier for cancer invasion. Consistent with their role in breast cancer progression, high levels of MMP-2 have been found to correlate with a poor prognosis in breast cancer patients (Talvensaari-Mattila *et al*, 1998; Duffy *et al*, 2000). These MMPs may also play a role in tumour vascularisation. Thus, it has been shown that the expression of MMP-2 correlates with VEGF and IGF-II expression, and that of MMP-9 with bFGF and increased microvessel density (MVD) (Kurizaki *et al*, 1998; Lee *et al*, 2000).

Previous studies have shown that antagonists of bombesin/gastrin-releasing peptide (GRP) inhibit the growth of various cancers by interfering with the growth-stimulatory effects of bombesin-like peptides, and reducing the levels of epidermal growth factor (Schally *et al*, 2001). Gastrin-releasing peptide mediates its action through specific membrane-bound receptors. The presence of bombesin/GRP receptors on the surface of breast cancer cells has been described (Giacchetti *et al*, 1990; De Jong *et al*, 1998a). In this regard, a significantly higher percentage of receptor-positive cases could be identified by autoradiography (Gugger and Reubi, 1999), compared with binding studies in cell lines, in which a 33% incidence of GRP receptor-positive breast carcinomas was found. In addition, our previous results revealed the expression of mRNA for only GRP receptor subtype 1 (GRPR) in MDA-MB-435 breast cancers (Bajo *et al*, 2002). Bombesin/GRP-like peptides bind these receptors, which are coupled to G proteins, activate protein kinase C and stimulate the release of Ca^2+^, finally leading to an increase in the expression of immediate-early genes such as *c-fos* and *c-jun*. Recently, we demonstrated that the treatment of MDA-MB-435 oestrogen-independent breast cancers with bombesin/GRP antagonists RC-3095 and RC3940-II decreased the expression of c-erbB-2/HER-2, as well as *c-jun* and *c-fos* oncogenes (Bajo *et al*, 2002). Therefore, the present study was performed to evaluate whether these antagonists can also affect the expression of the proangiogenic growth factors VEGF, FGF and IGF, the vessel density and the activities of MMP-2 and MMP-9, which play a basic role in the mechanisms of angiogenesis.

## MATERIALS AND METHODS

### Peptides

The bombesin/GRP antagonist D-Tpi^6^, Leu^13^*ψ*(CH_2_NH)Leu^14^-BN(6–14) (RC-3095), originally synthesised in our laboratory (Schally *et al*, 2001), was manufactured by ZENTARIS AG (Frankfurt am Main, Germany) in the form of acetate salt as D222213. The more modern antagonist Hca^6^, Leu^13^*ψ*(CH_2_N)Tac^14^-BN(6–14) (RC-3940-II) was synthesised and purified in our laboratory (Reile *et al*, 1995; Schally *et al*, 2001) (Hca is desaminophenylalanine, Tac is thiazolidine-4-carboxylic acid and Tpi is 2,3,4,9-tetrahydro-*H*-pyrido[3,4-b]indol-3-carboxylic acid). These peptides were dissolved in dimethyl sulphoxide (final concentration 0.1%) and diluted with 0.15 M sodium chloride in water for injection. Unless mentioned otherwise, all chemicals were purchased from Sigma (St Louis, MO, USA).

### Cell line and cell proliferation assays

Oestrogen-independent breast carcinoma cell line MDA-MB-435 was obtained from American Type Culture Collection (Manassas, VA, USA) and cultured in minimum essential medium (MEM) supplemented with 2 mM L-glutamine, 100 U ml^−1^ penicillin G sodium, 100 *μ*g ml^−1^ streptomycin sulphate, 0.25 *μ*g ml^−1.^ amphotericin B, 1 mM sodium pyruvate, 1 : 50 MEM vitamins, nonessential amino acids and 5% w v^−1^ foetal bovine serum. The cells were grown at 37°C in a humidified 95% air/5% carbon dioxide atmosphere, passaged weekly and routinely monitored for mycoplasma contamination using a detection kit (Boehringer Mannheim, Mannheim, Germany). All culture media components were purchased from Gibco (Grand Island, NY, USA). MDA-MB-435 cells were seeded into 96-well microplates (Falcon, Becton Dickinson and Co., Lincoln Park, NJ, USA). After 24 h, the culture medium was removed and replaced with serum-free medium (DMEM/F12, 1 : 50 MEM vitamins, 1 mM sodium pyruvate and 10 *μ*g ml^−1^ fetuin). GRP(14–27), RC-3095 or RC-3940-II were added to the medium at doses of 10^−8^ mol l^−1^. Controls received medium only. After an incubation period of 1 h, the medium was removed and kept at −70°C for RIAs. After an additional incubation in serum-free medium for 72 h, *in vitro* cell growth was estimated by the crystal violet assay (Bernhardt *et al*, 1992). The OD at 600 nm of each well was measured using a Beckman (Palo Alto, CA, USA) plate reader. Then, % *T*/*C* was calculated, where *T* is the optical density (OD_600 nm_) of treated cultures, and *C* is the OD_600 nm_ of control cultures × 100. The data presented are expressed as the mean±s.e. of eight replicate experiments. Each experiment was repeated two times, and similar results were obtained. For measurement of angiogenic factors in the media of MDA-MB-435 cells, samples were taken before seeding of cells (base medium) and immediately before treatments (t0).

### Animals

Female athymic (NCrnu/nu) nude mice (6-week old) were obtained from the National Cancer Institute (Frederic Cancer Research and Development Center, Frederick, MD, USA). The mice were housed in laminar airflow cabinets under pathogen-free conditions with a 12 h light/12 h dark schedule, and fed autoclaved standard chow and water *ad libitum*. All animal studies were conducted in accordance with institutional guidelines for the care, and were essentially in agreement with UKCCCR guidelines (1998) for the welfare of animals in experimental neoplasia.

### Experimental protocol

Tumours were initiated by orthotopic injection into the mammary fat pad of nude mice, as described previously (Bajo *et al*, 2002). The tumour volume was calculated using the formula: length × width × height × 0.5236. When tumours reached a volume of approximately 100 mm^3^, the mice were randomly divided into three experimental groups of 10 animals each, and received the following treatment as s.c. injections: group 1 (control), vehicle solution; group 2, antagonist RC-3095 at a dose of 20 *μ*g day^−1^ per animal; group 3, antagonist RC-3940-II at a dose of 10 *μ*g day^−1^ per animal. The treatment was continued for 42 days. At the end of the experiment, Metofane (Malinkrodt Vet, Mundelein, Il, USA) anaesthesia was used in overdose to kill the mice. Tumours were dissected, weighed and snap-frozen for further analyses.

### RNA extraction and reverse transcription–polymerase chain reaction (RT–PCR)

Total RNA was extracted from frozen tissue samples and cells by using RNAzolB (Tel-Test, Friendswood, TX, USA), according to the manufacturer's instructions. In all, 2–3 *μ*g of total RNA was reverse-transcribed into cDNA by Maloney murine leukaemia virus reverse transcriptase, according to the manufacturer's instructions (Perkin-Elmer Corp., Norwalk, CT, USA). For amplification of cDNA transcripts, gene-specific primers for human *β*-actin, VEGF-A, bFGF, IGF-I and -II were used as described in detail (Plonowski *et al*, 2000; Arencibia *et al*, 2001; Ueda *et al*, 2001). The number of cycles was determined in preliminary experiments to be within the exponential range of PCR amplification. Negative controls with water instead of cDNA were run in parallel to exclude genomic DNA contamination. PCR products were subjected to electrophoresis on a 1.5–2% agarose gel, then stained with ethidium bromide and visualised under ultraviolet light. Bands of PCR-amplified products were scanned and analysed semiquantitatively, using a zoom digital camera (DC290) with an EDAS 290 imaging system (Kodak, Rochester, NY, USA). All the experiments were repeated at least twice and similar results were obtained. The mRNA levels of each gene were normalised *vs* the corresponding levels of *β*-actin.

### Determination of VEGF-A, bFGF, IGF-I and -II concentration

VEGF-A, bFGF, IGF-I and -II levels were determined by radioimmunoassay (RIA) in extracts from tumour tissue and media from cultured cells. Tumours were homogenised in an ice-cold extraction buffer (50 mM Tris-HCl, pH 7.6 containing 1% v v^−1^ Triton X-100, 200 mM NaCl and 10 mM CaCl_2_). The chilled samples were stirred for 30 min and the supernatant was obtained by centrifugation (12 000 **g**, 20 min) and frozen at −70°C until use. VEGF-A, IGF-I and -II from tumour tissue and the media were extracted using a modified acid–ethanol cryoprecipitation method (Breier *et al*, 1991). The method eliminates most of the binding proteins, which can interfere with the RIA. The total protein content was determined using the BioRad protein assay kit (Bio-Rad Laboratories, Hercules, CA, USA).

The standard of VEGF was a recombinant human VEGF, 38.2 kDa, consisting of 165 amino acids. Anti-human VEGF was an affinity-purified, polyclonal antibody. Both VEGF and anti-human VEGF were purchased from PeproTech Inc (Rocky Hill, NJ, USA). The standard was used in the range of 0.006 ng and 12.8 ng tube^−1^. The antibody was used at a final dilution of 1 : 200 000. VEGF was iodinated by the lactoperoxidase method and purified by high-performance liquid chromatography, using a reverse-phase Vydac C8 column. The assay buffer for VEGF consisted of 0.01 M sodium phosphate pH 7.6, 0.025 M EDTA, 0.14 M NaCl, and 1% BSA. The antibody and tracer were added simultaneously and incubated overnight at 4°C. Bound and free fractions were separated by the polyethylene-glycol double-antibody method.

The standard of bFGF was a recombinant human growth factor, basic 17.2 kDa, consisting of 154 amino-acid residues (PeproTech Inc.). The standard curve ranged from 0.125 to 256 ng tube^−1^. The monoclonal antibody generated against bFGF, clone bFM-1, was purchased from Upstate Biotechnology (Lake Placid, NY, USA). The antibody was used at a final dilution of 1 : 20 000; it recognises the active conformation of bFGF molecule and is specific for bFGF from bovine, human and murine sources (Matsuzaki *et al*, 1989). [^125^I]Bolton Hunter-labelled FGF basic (human recombinant) was purchased from NEN-Perkin Elmer Life Sciences (Boston, MA, USA). The specific activity of [^125^I]bFGF was >1200 Ci mmol^−1^ (>70 mCi mg^−1^), and it was purified by affinity chromatography. The assay buffer consisted of 0.1% w v^−1^ gelatin–0.01 M phosphate pH 7.6, 0.025 M EDTA, 0.14 M NaCl, 0.05% v v^−1^ Tween-20 and 0.002 M dithiothreitol. The bound and free fractions were separated by the polyethylene-glycol double-antibody method.

Synthetic IGF-I (88-G4, Genentech, South San Francisco, CA, USA) was used as a standard in the range of 2–2000 pg tube^−1^, and also for iodination with the chloramine-T method. Rabbit anti hIGF-I (DSL Inc., Webster, TX, USA) was used at a final dilution of 1 : 10 000.

IGF-II concentration was measured by RIA using human recombinant IGF-II standard (Bachem, Torrance, CA, USA) in the range of 2–2000 pg tube^−1^. IGF-II was iodinated by the lactoperoxidase method, and purified as described previously (Lamharzi *et al*, 1998). Anti-rat IGF-II (10 *μ*g ml^−1^) monoclonal antibody (Amano International Enzyme, Troy, VA, USA) was used at a final dilution of 1 : 14 285. This antibody crossreacts 100% with human IGF-II and rat IGF-II, and 10% with human IGF-I.

The RIA results from all samples were evaluated in a computer-controlled gamma Counter (APEX Automatic Gamma Counter, Micromedic Inc., Huntsville, AL, USA). The interassay coefficients of variation of RIAs were less than 15%. The intraassay coefficients of variation were less than 10%.

### Zymography studies

Tumour extracts were examined by gelatin zymography. Samples were solubilised in nonreducing Laemmli buffer without heating, and electrophoresed at 4°C on 10% sodium dodecyl sulphate (SDS)–polyacrylamide gels containing 2 mg ml^−1^ gelatin (Sigma, St Louis, MO, USA). The gels were washed with wash buffer containing 50 mM Tris, pH 7.4 and 2.5% (v v^−1^) Triton X-100 twice for 30 min at 4°C. Triton-X was removed with wash buffer without Triton X-100, twice for 10 min. The gels were then incubated at 37°C for 18 h in an incubation buffer (50 mM Tris-HCl, pH 8.0, 5 mM CaCl_2_, 0.02% w v^−1^ sodium azide) and stained with 0.025% w v^−1^ Coomassie brilliant blue R-250. Both active forms and proenzymes could be revealed by this technique as the exposure of pro-MMPs to SDS during the electrophoresis leads to activation without proteolytic cleavage. The identification of the forms of MMP-2 and -9 was verified by comparison of the migratory position of the bands with known MMP-2 and -9 standards (Chemi-Con International, Temecula, CA, USA). Gelatinolytic activity appeared as a clear lysis zone within the blue background of the gelatin gel. These zones were analysed quantitatively using the imaging system specified above.

### Analysis of vessel density

Samples of tumour tissue were fixed in 10% buffered formalin and embedded in paraffin. Sections of 4 *μ*m thickness were cut and placed on silanated slides. Antigen retrieval was performed by placing the slides in 0.01 M sodium citrate buffer, pH 6.0 for 10 min at 95°C. After allowing the slides to cool for 20 min, endogenous peroxidase was quenched in 0.5% H_2_O_2_ for 10 min, followed by incubation in 1.5% w v^−1^ BSA for 1 h at room temperature. The slides were then incubated overnight at 4°C with the antibody directed against platelet endothelial cell adhesion molecule (PECAM)-1 (CD-31) (Santa Cruz Biotechnology, Santa Cruz, CA, USA) diluted 1 : 200. After repeated washing in 0.01 M sodium phosphate buffer (pH 7.4) containing 0.72% NaCl, Santa Cruz's ABC staining kit was used for detection. The slides were counterstained with 2% methyl green. MXT mouse mammary tumours, which are abundant in vessels, were used as positive controls. Negative controls were produced by omitting the primary antibody. For the determination of the vessel density (vascular area) in the tumours, the point-counting method based on Chalkley's principles was applied (Chalkley, 1943). An ocular net with 100 crossing points was used for the analysis. Tumour sections were scanned at a low power to identify the five most vascularised areas (hot spots) (Weidner *et al*, 1991). In these fields, the number of vessels that coincided with the crossing points of the net was counted at × 250 magnification, and the means of these numbers represented the % area of blood vessels in the slide of tumour tissue (Yamamura and Sato, 1974).

### Statistical analyses

Data are expressed as the mean±s.e. Statistical analyses were performed using Student's two-tailed *t*-test. All *P*-values are based on two-sided hypothesis testing. Histological data were evaluated by one-way analysis of variance, and the treated groups were compared with the control by Dunnett's test. *P*<0.05 was considered to be significant.

## RESULTS

### *In vitro* studies

**Figure 1:**
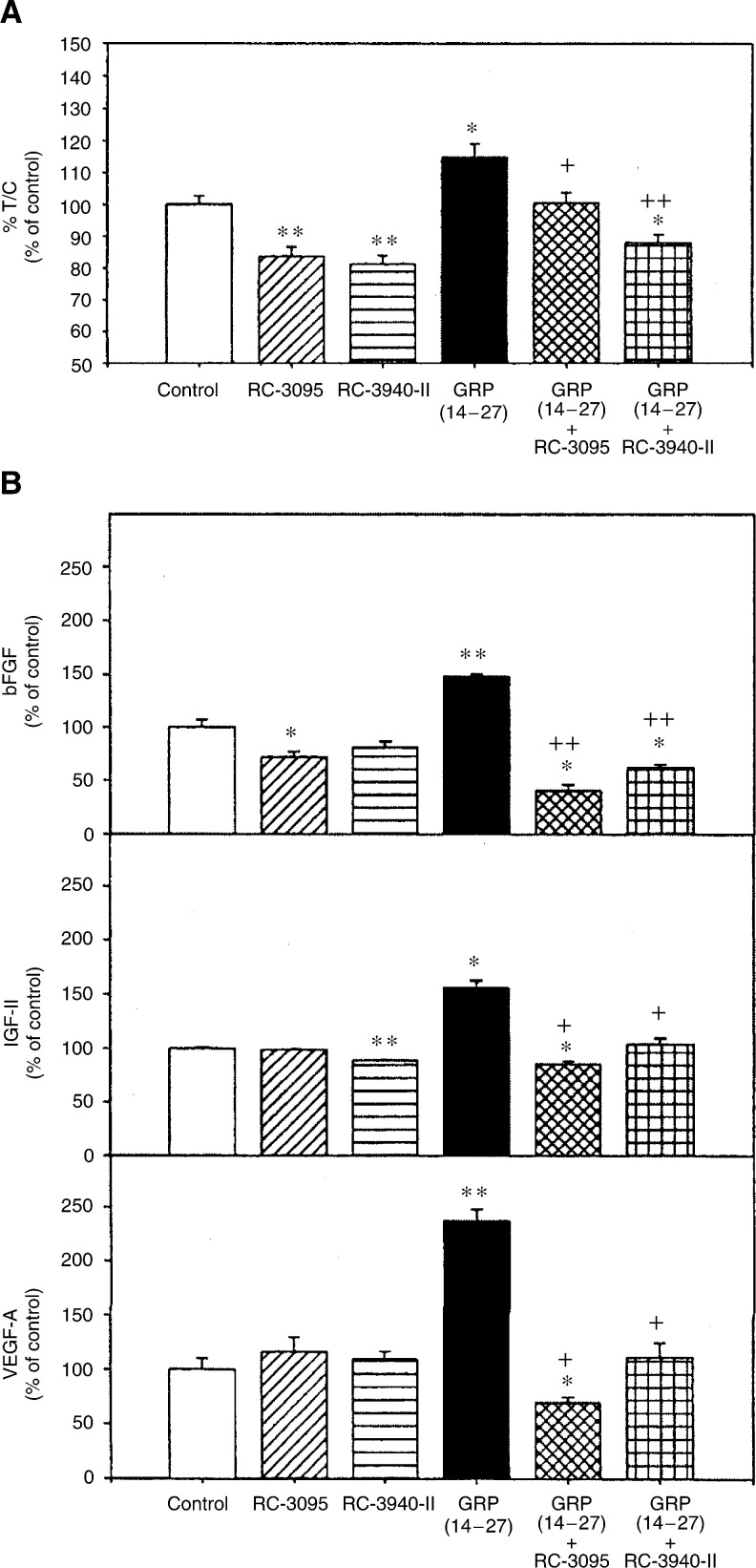
Effects of GRP(14–27) and/or bombesin/GRP antagonists (RC-3095 and RC-3940-II) at 10^−8^ mol l^−1^ for 1 h on the cell proliferation rate of MDA-MB-435 human breast cancer cells (**A**) and the concentration of bFGF, IGF-II and VEGF in medium (**B**). Growth was measured by crystal violet assay. The media from different wells of each treatment were collected and combined to perform RIA studies. Data are expressed as percentage of control. Vertical bars show s.e.'s; ^*^P<0.05 and ^**^P<0.005 *vs* control group; ^+^*P*<0.05 and ^++^*P*<0.005 *vs* GRP(14–27) group.

As shown in Figure 1a

, GRP(14–27) at a concentration of 10^–8^ mol l^−1^ produced about a 15% increase in the proliferation of MDA-MB-435 cells compared with the control value (*P*<0.05). The bombesin/GRP antagonists RC-3095 or RC-3940-II at concentrations of 10^–8^ mol l^−1^ significantly inhibited (*P*<0.005) the growth of MDA-MB-435 cells *in vitro* by 16 and 19%, respectively. When 10^−8^ M RC-3095 or RC-3940-1I were added together with GRP(14–27), the proliferation rate was significantly decreased by 13 and 23%, respectively, as compared with GRP(14–27) alone (Figure 1a). *In vitro* studies also showed that MDA-MB-435 cells produced and secreted bFGF (3–25 ng mg^−1^ of total protein), IGF-II (7–15 ng mg^−1^ of total protein) and VEGF-A (50–150 pg mg^−1^ of total protein) into the medium. GRP(14–27) significantly increased the concentration of bFGF, IGF-II and VEGF (*P*<0.05 and *P*<0.005) (Figure 1b). When GRP(14–27) was admixed with RC-3095 or RC-3940-II, the concentrations of bFGF, IGF-II and VEGF were significantly (*P*<0.05 and 0.005) decreased, as compared with those after GRP(14–27) treatment (Figure 1b).

### Effect of bombesin antagonists RC-3095 and RC-3940-II on tumour weights in nude mice with xenografted MDA-MB-435 breast cancers

**Figure 2:**
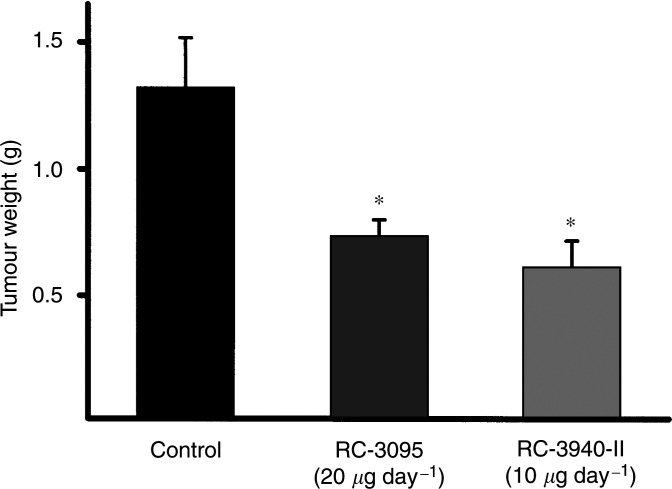
Tumour weights of MDA-MB-435 human oestrogen-independent breast carcinomas, implanted orthotopically into athymic female nude mice, after treatment with bombesin/GRP antagonists RC-3095 or RC-3940-II. Vertical bars show s.e.'s; ^*^P<0.05 *vs* control, two-tailed *t*-test.

Both antagonists reduced the weight of MDA-MB-435 tumours in nude mice (Figure 2

). After 42 days of treatment, RC-3095 administered at 20 *μ*g day^−1^ per animal significantly (*P*<0.05) reduced the mean tumour weight to 0.73±0.06 g compared with that in the control group (1.32±0.19 g), corresponding to a decrease of about 44.3%. An even greater reduction in tumour weight (53.6%, *P*<0.05) was observed in mice treated with RC-3940-II at 10 *μ*g day^−1^ per animal.

### mRNA analyses for bFGF, IGF-I and -II and VEGF-A

**Figure 3:**
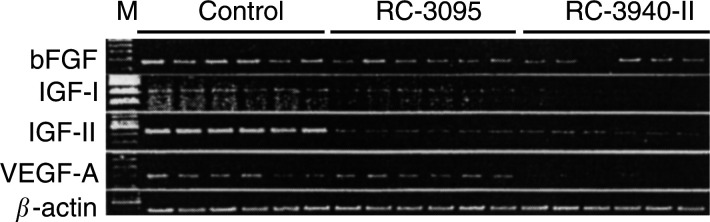
Expression of mRNA for bFGF, IGF-I and -II and vascular endothelial growth factor-A (VEGF-A) in MDA-MB-435 human oestrogen-independent breast cancer grown in nude mice after treatment with RC-3095 (20 *μ*g day^−1^) or RC-3940-II (10 *μ*g day^−1^). The total RNA (2–3 *μ*g) was reverse transcribed and cDNA was amplified with specific primers. The PCR products were resolved on 1.5–2% agarose. mRNA expression for *β*-actin was used as an internal control. PCRs yielded products of the expected size of 270, 514, 538, 201 and 459 bp for the bFGF, IGF-I and-II, VEGF-A and *β*-actin, respectively. M, l00-bp molecular DNA marker. The figure shows six representative samples of each group.

To investigate the effect of the treatment with bombesin/GRP antagonists on the expression of angiogenic factors such as bFGF and IGF-I and -II and VEGF-A, RT–PCR analysis was performed. PCR products of 270, 514, 538 and 201 bp, corresponding to the bFGF and IGF-I and -II and VEGF-A, respectively, were detected after ethidium bromide staining (Figure 3

). No amplified PCR products were obtained from the negative controls, ruling out the possibility of genomic DNA contamination (data not shown). The amplification with *β*-actin-specific primers produced a single product of 459 bp from all samples, confirming that there was no degradation of the RNA preparations. Densitometric analyses of the RT–PCR products are shown in Table 1

**Table 1:** Effect of treatment with RC-3095 and RC-3940-II on mRNA expression and protein levels of bFGF, IGF-I and -II and VEGF in MDA-MB-435 human breast carcinomas

	**mRNA (% of control)^a^,^b^**			**Protein (% of control)^a^,^b^**		
	**Control**	**RC-3095**	**RC-3940-II**	**Control**	**RC-3095**	**RC-3940-II**
bFGF	100.0±8.3	60.5±9.1^d^	63.4±9.3^c^	100.0±13.8	62.9±7.3^c^	55.3±6.0^d^
IGF-I	100.0±11.5	73.9±7.5	71.8±6.0	Detection limit	Detection limit	Detection limit
IGF-II	100.0±3.9	22.7±2.1^d^	23.5±1.6^d^	100.0±18.8	64.3±8.0^d^	75.4±5.5^c^
VEGF	100.0±4.1	87.0±2.3^c^	63.7±6.4^d^	100.0±6.3	90.0±15.2	50.9±9.5^d^

a Mean±s.e.

b mRNA levels were standardised according to *β*-actin mRNA levels, and are expressed as percentage of control value. Protein levels were determined by RIA, as described in ‘Materials and methods’.

c *P*<0.05 *vs* control, two-tailed *t*-test.

d *P*<0.01 *vs* control, two-tailed *t*-test.

. After normalisation of the expression of mRNA for the angiogenic factors *vs* the corresponding *β*-actin mRNA levels in MDA-MB-435 tumours, RC-3095 was found to diminish mRNA for bFGF, IGF-II and VEGF-A, by 39.4% (*P*<0.01), 77.3% (*P*<0.01) and 13% (*P*<0.01), respectively. RT–PCR analyses also revealed that treatment with RC-3940-II decreased the expression of mRNA for bFGF, IGF-II and VEGF-A by 36.6% (*P*<0.05), 76.1% (*P*<0.01) and 36.3% (*P*<0.05), respectively (Table 1). In addition, there was a reduction (not significant) in the expression of mRNA for IGF-I in both treated groups as compared with the control (Table 1).

### Radioimmunoassays for VEGF-A, bFGF and IGF-I and -II in MDA-MB-435 tumours

To demonstrate that the decrease in the expression of VEGF-A, bFGF, IGF-II mRNA induced by RC-3095 and RC-3940-II resulted in changes in the corresponding protein levels, tumour extracts were evaluated by RIA. Protein levels of bFGF, IGF-II and VEGF-A in MDA-MB-435 cancers from animals treated with RC-3940-II showed a 44.7% (*P*<0.01), 24.6% (*P*<0.05) and 49.1% (*P*<0.01) decrease, respectively (Table 1). RC-3095 significantly reduced bFGF by 37.1% (*P*<0.05) and IGF-II by 35.7% (*P*<0.01), and caused a slight and nonsignificant decrease in VEGF-A protein levels (Table 1). IGF-I levels were at the detection limit of the RIA.

### Detection of gelatinolytic activity

**Figure 4:**
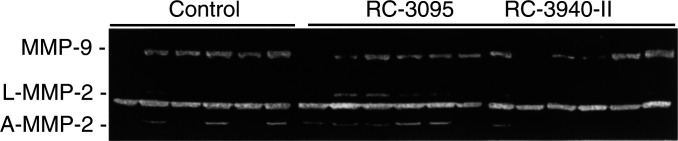
Gelatin zymography of extracts from MDA-MB-435 tumours treated with bombesin antagonists RC-3095 and RC-3940-II at doses of 20 and 10 *μ*g day^−1^ animal^−1^, respectively, for 6 weeks. Total protein (10 *μ*g) from tumour extracts was subjected to zymography, as described in Materials and methods. The figure shows six representative samples from each group. The positions of the latent (L) and activated (A) forms of MMP-2 and latent MMP-9 are indicated on the left, and were identified as described in the text.

Gelatinolytic activities with different molecular masses were detected in MDA-MB-435 tumour extracts from control and treated animals by gelatin zymography (Figure 4

). Two bands migrating in the 60–67 kDa molecular mass range were seen, indicating the upper and lower bands of lysis as the latent and activated forms of MMP-2 (labeled L and A), respectively. The presence of a band migrating at 96 kDa, which represents the latent form of MMP-9, is also shown in Figure 4. Densitometric analyses of the gelatinolytic activities of both enzymes in MDA-MB-435 tumours treated with RC-3095 (20 *μ*g day^−1^) and RC-3940-II (10 *μ*g day^−1^) are shown in Table 2

**Table 2:** Effects of bombesin antagonists RC-3095 and RC-3940-II on the gelatinolytic activity of MMP-2 and MMP-9 in MDA-MB-435 breast cancers

	**Control^a^**	**RC-3095 (% of control)^a^**	**RC-3940-II (% of control)^a^**
Active MMP-2	100.0±11.4	104.5±12.4	63.4±7.5^b^
Latent MMP-2	100.0±6.9	104.0±8.6	99.7±8.9
Latent MMP-9	100.0±10.2	51.4±5.3^c^	67.0±10.4^b^

a Mean±s.e.

b *P*<0.05 *vs* control, two-tailed *t*-test.

c *P*<0.01 *vs* control, two-tailed *t*-test.

. The active form of MMP-2 was not affected by RC-3095, but was significantly (*P*<0.05) reduced in the group receiving RC-3940-II to 1.23±0.1 arbitrary units (a.u.), compared with the control group, which amounted to 1.94±0.2 a.u., corresponding to a decrease of about 40%. The same gelatinolytic activities from latent MMP-2 isoform were observed in the tumours from mice treated with either antagonist. The latent form of MMP-9 was significantly decreased in the groups treated with RC-3095 (*P*<0.01) and RC-3940-II (*P*<0.05) to 5.3±0.5 and 6.9±1.0 a.u., corresponding to reductions of 48.6 and 33.0%, respectively, as compared with the control group, which came to 10.3±1.0 a.u.

### Analysis of vessel density

**Figure 5:**
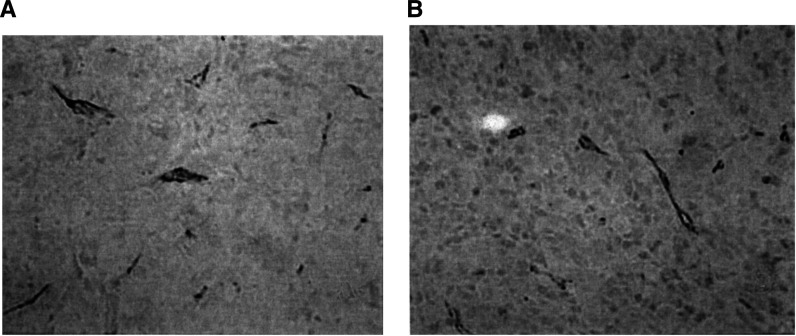
Representative ‘vascular hot spots’ in MDA-MB-435 cancers demonstrated by immunohistochemical detection of CD-31 antigen in endothelial cells, as described in ‘Materials and methods’. (**A**) A control tumour and (**B**) tumour treated with RC-3940-II. Methyl green counterstain, × 180.

Immunohistochemical detection of CD-31 antigen in endothelial cells showed the vascular characteristics of MDA-MB-435 tumours well (Figure 5

). Only intratumoral vessels surrounded by living tumour cells were considered for measurement. The vascular hot spots were mostly found close to the surface of the tumour. The vessel density of the tumours was significantly (*P*<0.05) decreased by RC-3095 to 3.92±0.4% and by RC-3940-II to 3.89±0.5%, corresponding to a reduction of more than one-third as compared with the control group, which had a vessel density of 6.18±0.9%.

## DISCUSSION

The present methods for the treatment of oestrogen-independent breast cancers are not very effective and new therapeutic modalities must be developed (Schally and Comaru-Schally, 2003). The treatment with our bombesin/GRP antagonists RC-3095 and RC-3940-II results in a significant reduction in the growth of MDA-MB-435 human mammary cancers in nude mice, as reflected by tumour weights. A similar inhibition of growth of human H-69 small cell lung cancer, ES-2 and OV-1063 ovarian cancers, MDA-MB-231 and MDA-MB-468 breast cancers, PC-3 and DU-145 prostate cancers, CAKI-1 renal cell carcinomas, U-87MG glioblastomas, SW-1990 pancreatic cancer and other tumours induced by RC-3095 or RC-3940-II was reported previously (Schally *et al*, 2001). The present study demonstrates that the inhibition of angiogenesis could be one of the mechanisms of action of the bombesin/GRP antagonists.

Numerous reports indicate that growth factors such as VEGF, bFGF, IGF-I and -II are involved in the angiogenic process in various tumours including breast cancer (Kandel *et al*, 1991; Grant *et al*, 1993; Heffelfinger *et al*, 1999; Neufeld *et al*, 1999; Lee *et al*, 2000; Yan *et al*, 2000). Our results demonstrate that the treatment of MDA-MB-435 oestrogen-independent breast cancers with bombesin/GRP antagonists RC-3095 and RC-3940-II significantly decreased the expression of mRNA for VEGF-A, b-FGF and IGF-II, as well as their protein levels. A similar inhibition of IGF-II expression by RC-3940-II has been observed in PC-3 human prostate cancer (Plonowski *et al*, 2000).

*In vitro* studies show that exogenous GRP(14–27) enhances the growth of MDA-MB-435 cells in serum-free medium, and bombesin/GRP antagonists RC-3095 and RC-3940-II inhibit this proliferation. Similar effects on the cell growth of breast, brain, pancreatic and gastric cancers were reported previously (Schally *et al*, 2001). We also showed that MDA-MB-435 cells produce and secrete bFGF, IGF-II and VEGF-A. GRP(14–27) stimulates the secretion of bFGF, IGF-II and VEGF-A from MDA-MB-435 cells, but either antagonist can abolish this stimulation. This may suggest a receptor-based mechanism for tumour growth inhibition, and supports the hypothesis of direct action of RC-3095 and RC-3940-II on MDA-MB-435 cells. The presence of these factors as well as their receptors on plasma membranes corroborates an autocrine loop previously described in breast cancer (De Jong *et al*, 1998a).

Recent findings show the inter-relationship of the angiogenic growth factors and the MMPs in human breast carcinomas. Thus, there is a significant correlation between activated MMP-2 expression and VEGF expression, and similarly pro-MMP-9 expression tends to correlate with the increment of MVD (Kurizaki *et al*, 1998). IGF-II has also been described as an angiogenic factor that acts either directly (Volpert *et al*, 1996; Lee *et al*, 2000) or indirectly through an increase in VEGF production and the upregulation of the expression of proteolytic enzyme MMP-2 (Kim *et al*, 1998). Overactive MMPs contribute to an almost complete loss of the peritumoral basement membrane in most cancers including breast carcinoma (Lebeau *et al*, 1999). In addition, bFGF expression has shown a positive correlation with the mytotic activity index (De Jong *et al*, 1998b). Furthermore, the overexpression of ErbB-2/HER-2, an important predictive factor in breast cancer, is also closely associated with increased angiogenesis (Yen *et al*, 2000). Thus, the anti-HER-2 blocking antibody trastuzumab (herceptin) acts as an antiangiogenic ‘cocktail’ controlling the expression of various proangiogenic factors including VEGF, TGF-*α*, Ang-1 and PAI (Kumar and Yarmand-Bagheri, 2001; Izumi *et al*, 2002). In this regard, previous studies have shown that Gq-coupled receptors such as those for bombesin/GRP can phosphorylate receptors of the ErbB/HER family by transactivation (Prenzel *et al*, 1999). It has also been revealed that EGFR and HER-2 can potentiate tumour cell adhesion to endothelial cells, by enhancing the synthesis of hypoxia-inducible factor (HIF)-1*α*, and/or carbonic anhydrase-9 (Giatromanolaki *et al*, 2001; Laughner *et al*, 2001) through a PI3 kinase-dependent pathway (Bagheri-Yarmand *et al*, 2000). Several dozens of HIF-1 targets are known, including the genes encoding VEGF and IGF-II. The relationship of HIF-1 with bFGFR has also been described (Kim *et al*, 1998; Semenza, 1999; Giatromanolaki *et al*, 2001; Laughner *et al*, 2001).

It is interesting that bombesin antagonists affect MMP-9 differently from MMP-2. Previous studies have shown that bombesin/GRP-like peptides activate the dimeric AP-l complex composed of Jun and Fos proteins, which bind to the AP-l element and activate the transcription (Beno *et al*, 1995). In our investigation, the activated MMP-2 isoform expression was decreased after treatment with bombesin/GRP antagonist RC-3940-II. However, the latent isoform was not affected by the antagonists used. This result could indicate that such antagonists would not affect the expression of MMP-2 at the transcriptional level, but may control elements involved in its proteolytic activation. On the other hand, the latent MMP-9 isoform was downregulated by both antagonists. One possible explanation for the finding that bombesin/GRP antagonists affect MMP-2 and MMP-9 differently is that MMP-2 does not contain AP-l-binding sites in its promoter, but MMP-9 gene harbours an AP-1-binding site in the proximal promoter (Nagase and Woessner, 1999). The changes in angiogenic growth factors and MMPs caused by the bombesin/GRP antagonists were also accompanied by a significant reduction in the vessel density of MDA-MB 435 breast tumours. This is in accord with earlier findings that the expression of pro-MMP-9 and the microvascular density may be related in human breast carcinoma (Kurizaki *et al*, 1998).

In conclusion, the present study demonstrates that bombesin/GRP antagonists reduce the expression of mRNA and protein levels of the most significant proangiogenic factors in breast cancer. These angiogenic and growth-promoting substances closely interact with each other. The tumour-inhibitory effect of bombesin/GRP antagonists appears to involve complex mechanisms. In this regard, we recently reported that RC-3095 and RC-3940-II can inhibit the growth of MDA-MB-435 cancer by downregulating mainly ErbB-2/HER-2, as well as by decreasing the expression of *c-jun* and *c-fos* oncogenes (Bajo *et al*, 2002). Therefore, the mechanism responsible for the action of these antagonists on angiogenesis could be mediated by a downregulation of ErbB-2/HER-2, which would affect the pathways described above, and finally facilitate a decrease in the expression of the growth factors and MMPs as well as the vascular content. Future studies will be aimed at the clarification of the signalling pathways related to the antitumour effect of RC-3095 and RC-3940-II in HER-2-positive breast carcinomas. Our findings confirm the merit of continued investigations of bombesin/GRP antagonists for the development of another approach to the management of breast cancer.
